# Betaine Supplementation Into High-Carbohydrate Diets Improves Feed Efficiency and Liver Health of *Megalobrama amblycephala* by Increasing Taurine Synthesis

**DOI:** 10.1155/2024/9632883

**Published:** 2024-09-26

**Authors:** Wenbo Pan, Fan Wang, Jia Xu, Juntao Li, Jian Gao, Yuhua Zhao, Qingchao Wang

**Affiliations:** ^1^ College of Fisheries Key Lab of Freshwater Animal Breeding Ministry of Agriculture Huazhong Agricultural University, Wuhan 430070, China; ^2^ Guangxi Key Laboratory of Marine Environmental Science Guangxi Academy of Marine Sciences Guangxi Academy of Sciences, Nanning 530012, China; ^3^ Institute of Tropical Bioscience and Biotechnology Hainan Institute for Tropical Agricultural Resources Chinese Academy of Tropical Agricultural Sciences, Haikou 570102, China

**Keywords:** betaine, high-carbohydrate, *Megalobrama amblycephala*, taurine

## Abstract

Dietary betaine supplementation has been reported to alleviate the adverse effects of high-carbohydrate diets on *Megalobrama amblycephala*, while the regulatory mechanism remains largely unknown. In the present study, a 79-day feeding trial was conducted with 450 juvenile *Megalobrama amblycephala* (average weight 6.75 ± 0.10 g), which were fed with five high-carbohydrate diets (43%) supplementing betaine at 0% (CD group), 0.2% (0.2Bet group), 0.4% (0.4Bet group), 0.8% (0.8Bet group), and 1.6% (1.6Bet group), respectively. Results showed *M. amblycephala* in 0.8Bet group exhibited the best growth performance, indicated by the largest weight gain ratio (142.88%) and least feed conversion ratio (1.63). Moreover, liver health was promoted in 0.8Bet group, with decreased number of non-nucleated cells and less lipid accumulation, which was accompanied by the lowest hepatosomatic index (1.38%). In order to further illustrate the regulatory mechanism, metabolites assay indicated that dietary betaine supplementation significantly increased plasma contents of methionine, serine, hypotaurine, and taurine, but did not affect plasma contents of cystathionine, cystine, or cysteic acid. Accordingly, the mRNA expressions of cysteine sulfinate decarboxylase in cysteine sulfinic acid pathway and cysteamine dioxygenase (ADO) in sulfinic acid (CS) pathway, which were both involved in taurine synthesis, were also upregulated in the liver. Meanwhile, the microbial communities in *M. amblycephala* intestine were more stable and uniform with betaine supplementation. Therefore, dietary betaine supplementation may exert its protective roles in improving feed efficiency and liver health of *M. amblycephala* via promoting de novo taurine synthesis and stabilizing intestinal microbial communities.

## 1. Introduction

*Megalobrama amblycephala*, a freshwater fish known for its economic value [1], reached a production of 781.7 thousand tons worldwide in 2020 [2]. Sustainable aquaculture depends on balanced feed formulations, with protein sources being a key constraint. Carbohydrates, abundant and cost-effective, serve as the primary energy source in aquatic feeds [3, 4], exerting a protein-sparing effect within a specific range [5]. However, excessive carbohydrates can adversely affect growth, metabolism, and health in various fish species [6–8]. High-carbohydrate diets may lead to metabolic disturbances, stress responses, hyperglycemia, and lipid deposition [3, 6, 9–11]. Even herbivorous fish like *M. amblycephala* are not immune to the negative effects of high-carbohydrate diets on liver health [4, 12, 13]. Therefore, developing functional additives to mitigate these effects is crucial.

Betaine, derived from the amino acid glycine, is widely present in microorganisms, marine life, plants, and animals [7, 14]. In animals, betaine acts as a methyl group donor, organic osmolyte, and plays other important biological roles [15]. Due to its distinctive flavor, betaine is commonly used as an effective attractant in aquafeed [16–18]. Recent studies have shown that dietary betaine supplementation improves fish growth, survival rates, feed utilization, and aquaculture costs [19–21]. In species like rainbow trout and *Salmo trutta*, betaine supplementation enhances feed acceptability, growth performance, immune response, antioxidant defense, and digestive activity [22, 23]. In *M. amblycephala*, betaine supplementation reduces hepatic lipid accumulation and reprograms bile acid and trimethylamino-*N*-oxide metabolism [13]. Betaine also improves fish immunity, regulates intestinal flora, and maintains osmotic pressure [24, 25], suggesting its potential in promoting growth and reducing liver damage induced by high-carbohydrate diets.

Previous metabolomics studies have shown that both betaine and taurine levels decrease significantly in *M. amblycephala* plasma fed with high-carbohydrate diets [20], indicating a strong correlation between betaine and taurine. Betaine can facilitate the formation of cysteine, which in turn promotes taurine synthesis [25–27]. Taurine levels are low in obese and diabetic individuals, and taurine supplementation can delay obesity progression in mice fed high-fat diets [28, 29]. Dietary taurine supplementation in various fish species reduces whole-body and liver lipid content by affecting bile acid synthesis [30–33]. Considering the association between betaine and taurine, and taurine's role in lipid reduction, it is hypothesized that betaine may protect *M. amblycephala* by enhancing endogenous taurine synthesis. A study feeding *M. amblycephala* with high-carbohydrate diets supplemented with varying levels of betaine for 79 days aims to determine the optimal supplementation level based on growth performance and liver health. Additionally, the study will analyze serum taurine-related metabolite content, hepatic taurine biosynthetic gene expression, and intestinal microbial communities to elucidate the regulatory mechanism.

## 2. Materials and Methods

### 2.1. Diet Preparation, Feeding Experiment, and Sample Collection

*M. amblycephala* has been reported to exhibit stress response when fed diets with dietary carbohydrate over 31% (31%–47%) [34], thus a control diet was formulated to contain over 43.47% of carbohydrates (CD, 0% betaine addition). Based on this, four experimental diets with detaine addition were designed as follows: 0.2% betaine addition (0.2B), 0.4% betaine addition (0.4B), 0.8% betaine addition (0.8B), and 1.6% betaine addition (1.6B) (Table 1). Diet preparation, storage and analysis methods, along with fish acclimation followed the same procedures as reported by Prisingkorn et al. [4]. Betaine and bentonite were added in the mixing process of raw material powder. In order to ensure the uniform distribution of betaine in the diets, betaine was melted into water and uniformly sprayed onto the surface of the raw materials using a sprayer during the mixing process of feed raw materials. Crude protein, crude fat, moisture, and crude ash composition of diets (feeds) were determined by standard methods exactly as previously described [4]. Carbohydrate (nitrogen-free extract) content of diets was analyzed by the 3,5-dinitro salicylic acid method [35].

Healthy juvenile fish (average weight, 6.75 ± 0.10 g) were obtained from wet lab of Huazhong Agricultural University, Wuhan, China, and randomly distributed into 15 plastic tanks (30 fish/tank) after weighing with three replicate tanks randomly assigned for each experimental diet group (CD, 0.2B, 0.4B, 0.8B, and 1.6B). Fish were fed twice daily with the ratio at 2.0%–3.0% of their body weight, which was regularly adjusted according to feeding performance. The amount of feed consumed and fecal matter excreted in each tank were recorded twice daily. Water temperature, oxygen content, nitrite, and total ammonia nitrogen (TAN═NH_3_–N+NH^4+^–N) in the tanks were controlled to be 24 ± 2°C, > 8 mg O_2_/L, <0.1 mg NO^2−^/L, and <0.1 mg TAN/L, respectively.

At the end of 79 days feeding trial, fish were anesthetized with 100 mg/L MS-222 (Sigma Aldrich, USA) after a 24-h fasting period. Fish weight, body length, and width were measured. Then, 15 fish in each tank were selected randomly to obtain the blood, liver, muscle, and gut microbiota samples. Blood was obtained from the caudal vein using sterile syringes with preadded anticoagulant solution and then centrifuged (3000 r/min) at 4°C for 10 min to obtain the plasma, which was quickly frozen in liquid nitrogen and stored at −80°C for biochemical assays. After euthanizing the fish, each liver was weighed and divided into two parts: one was placed in formalin for histological examination, and the remainder was quickly frozen in liquid nitrogen and stored at −80°C for quantitative real-time PCR (qRT-PCR). Intestinal contents were collected into a tube filled with lumen wash solution, then gently agitated at 4°C for 10 min, followed by centrifugation (12,000 r/min) for 5 min, and finally, the supernatant was stored at −80°C for microbial DNA extraction. All animals were handled and experimental procedures were conducted in accordance with the guidelines for the care and use of animals for scientific purposes set by the Ministry of Science and Technology, Beijing, China (No. 398, 2006).

### 2.2. Growth Performance, H&E Staining, and Biochemical Analysis

The following growth parameters were calculated based on the data of 15 fish collected per tank: weight gain (WGR), specific growth rate (SGR), feed conversion ratio (FCR), hepatosomatic index (HSI), and condition factor (CF):

(1) $$\begin{array}{c} \text{Weight gain rate }\left(\text{WGR},\%\right)=\left(\text{Final body weight –initial body weight}\right)\times 100/\text{Initial body weight}, \end{array}$$

(2) $$\begin{array}{c} \text{Specific growth rate }\left(\text{SGR},\%/\text{day}\right)=100\times \left(\text{Ln final body weight –ln initial body weight}\right)/\text{Experiment duration in days}, \end{array}$$

(3) $$\begin{array}{c} \text{Feed conversion ratio }\left(\text{FCR}\right)=\text{Dry diet fed}/\text{Wet weight gain}, \end{array}$$

(4) $$\begin{array}{c} \text{Hepatosomatic index }\left(\text{HSI},\%\right)=100\times \text{liver wet weight}/\text{body wet weight}, \end{array}$$

(5) $$\begin{array}{c} \text{Condition factor }\left(\text{CF},\text{ g}/{\text{cm}}^{3}\right)=100\times \text{body weight}/\left({\text{body length}}^{3}\right). \end{array}$$

The liver tissues of three fish (nine in each group) were selected from each tank for fixation, dehydration, and paraffin embedding. Liver sections (3 μm thickness) were then subjected to hematoxylin and eosin (H&E) staining. Biochemical determination of liver tissue of three fish (nine in each group) was selected from each tank. Alanine aminotransferase Assay (ALT) Kit (No. C009-2-1), aspartate aminotransferase assay (AST) Kit (No. C010-2-1) and triglyceride assay (TG) Kit (No. A110-1-1) from Nanjing Jiancheng Bioengineering Institute (China) was used to measure plasma ALT, AST content, and liver TG content.

### 2.3. Analyses of Metabolites and Amino Acids Related to Taurine Synthesis

Taurine in plasma was detected by a high-performance liquid chromatography-tandem mass spectrometry (HPLC-MS/MS) on the Ultimate 3000 (Dionex, Sunnyvale, USA)-API 3200Q TRAP (AB Sciex, Framingham, MA, USA) system, three fish were detected in each tank (nine fish in each group). The analyses were performed by the Beijing Mass Spectrometry Medical Research Co., Ltd. (Beijing, China). In brief, 50 μL of protein precipitant (containing NVL) was added to 50 μL plasma, mixed thoroughly, and centrifuged. The supernatant (10 μL) was mixed with 50 μL of methanol borate buffer thoroughly, the above solution was mixed with 20 μL of diethyl ethoxymethylene-malonate. This solution (50 μL) was analyzed by HPLC-MS/MS. Chromatographic separations were performed on an MS Lab C18 column (150 × 4.6 mm, 5 μm). Mobile phase A was 0.1% formic acid in water (v/v), and the organic mobile phase B was 0.1% ammonium formate in acetonitrile (v/v) with pH 5.8. Solvent was delivered to the column at a flow rate of 1 mL/min. Calibration solution was used for calibration of the system and quantification of metabolites. Data were processed using Analyst software Version 1.5.1 (Applied Biosystems).

The plasma of three fish (nine in each group) was selected from each tank for quantitative analysis of amino acids. Quantification of amino acids was performed by amino acid analyzer A300 (MembraPure GmbH, Germany). The plasma solution in 4 volumes of samples was precipitated by adding 1 volume of 10% (w/v) sulfosalicylic acid solution, and then the mixtures were incubated for 1 h at 4°C and centrifuged (14,500 r/min, 15 min, 4°C). The supernatant was centrifuged again (14,500 r/min, 5 min, 4°C), then filtered through Nylon66 membrane filter with a 0.22-μm pore size (Ameritech Scientific). Amino acids were separated by liquid chromatography on an ion-exchange column (Li-separation column, membra-Pure) and revealed by the ninhydrin reaction, followed by absorbance measurements at 570 nm. The concentration of each amino acid was calculated using external standards (Sigma–Aldrich, St. Louis, MO, USA) and expressed in nmol/mL.

### 2.4. qPCR Analysis

Three fish (nine fish in each group) were selected from each tank for qPCR analysis. Genes (enzymes) associated with taurine metabolism and fatty metabolism were selected in the KEGG database [36] for expression pattern analyses: cysteamine dioxygenase a and b (ADOa and ADOb), cysteine dioxygenase 1 (CDO1), cysteine sulfinate decarboxylase (CSAD), glutamate decarboxylase (GAD), fatty acid synthase (FAS), acetyl COA carboxylase (ACC), Diacylglycerol acyltransferase 2 (DGAT2), and acyl COA oxidase 1 (ACOX1). Primers for these genes (Table 2) were designed by Primer Premier 5 software (Premier Biosoft, USA) on the basis of the whole genome data [37] and synthesized by the Shanghai Sheng Gong Co. *β*-actin (F: CGGACAGGTCATCACCATTG; R: CGCAAGACTCCATACCCAAGA) was selected as the reference gene on the basis of its expression stability [1]. Total RNA isolation, cDNA preparation, and qPCR detection of each sample were performed in full accordance with the previous description [4], with the only difference that each sample was analyzed in quadruplicate.

### 2.5. T-RFLP Analysis in Gut Microbiota

Gut contents were collected from 15 fish specimens in each tank and mixed for 16S rRNA sequencing, and there were three repetitions in each group. Genomic DNA of gut microbiota was obtained using the hexadecyltrimethylammonium bromide extraction method. The extracted DNA was quantified spectrophotometrically with NanoDrop 2000 spectrophotometer (Thermo Scientific, Delaware, USA) at A260/280 and diluted to 25 ng/μL. The 6-carboxyfluorescein (6-FAM) labeled 27 f forward primer and nonlabeled 1492r reverse primer were used for the amplification of 16S rRNA fragments of extracted DNA (27 F: AGAGTTTGATCMTGGCTCAG, 1 492R: TACGGYTACCTTGTTACGACT). Digestion reactions (25 μL) were carried out with 500 ng of purified PCR products and 25 U of the *HaeIII* enzyme restriction endonuclease (NEB Biotechnology, Beijing, China), and *MspI* enzyme digestion reactions were carried out in the same way. The resultant digestion products, containing fluorescently labeled terminal restriction fragments (T-RF), were analyzed on the ABI 3730XL DNA analyzer (Applied Biosystems Instruments, Foster City, CA). Based on the T-RF lengths and peak heights [38], we evaluated changes in the microbial community composition using the following indices: Shannon (diversity of a biological community), Evenness (quantifying how equal the community was numerically) [39], and Simpson (species diversity within an ecological habitat). Principal component analysis (PCA) and cluster analysis were adopted to infer the similarities and differences in the T-RF composition.

### 2.6. Statistical Analysis

Data were organized and analyzed by Microsoft Excel 2016 and SPSS 19 (SPSS, Chicago, USA), statistical analysis was performed on all sample data of each group as a whole. The calculation formulas of Shannon–Weiner index (H′), Simpson index (*D*′), and Evenness index (*E*′) during the T-RFLP of microorganisms are as follows: *H*′ = −∑*pi* ln *pi*; *D*′ = 1 − ∑*pi*^2^; and *E*′ = *H*′/*H*_max_, (*pi* is the ratio of the peak height of the *i*th T-RF to the maximum peak height of the population, and *H*_max_ is the natural logarithm of the number of T-RFs). PCA and cluster analysis of T-RFs were performed by SPSS 19 and MEGA 7, respectively. After statistical analysis, all data were in accordance with the normal distribution and passed the homogeneity test of variance. Data of growth performance, plasma metabolites, and microbial community composition indices were subjected to one-way analysis of variances (ANOVA). Significant differences among the group means were further compared using Tukey's multiple range tests at the *p* < 0.05 threshold of statistical significance. Results of plasma taurine content and gene expression were analyzed by *t*-test, and significance thresholds were set at *p* < 0.05 (significantly).

## 3. Result

### 3.1. Growth Performance

As shown in Table 3, the weight gain rate (WGR) and specific growth rate (SGR) of 0.8B group and 1.6B group were significantly higher than those in CD group and 0.2B group (*p* < 0.05). Moreover, the feed conversion ratio (FCR) in 0.8B group was the lowest, which was significantly lower than that of CD group and 0.2B group (*p* < 0.05). The HSI in 0.4B group and 0.8B group was significantly lower than that of the other three groups. Thus, dietary betaine supplementation improved fish growth parameters and blunt snout bream in 0.8B group showed the best growth performance.

### 3.2. Liver Health

**Figure 1:**
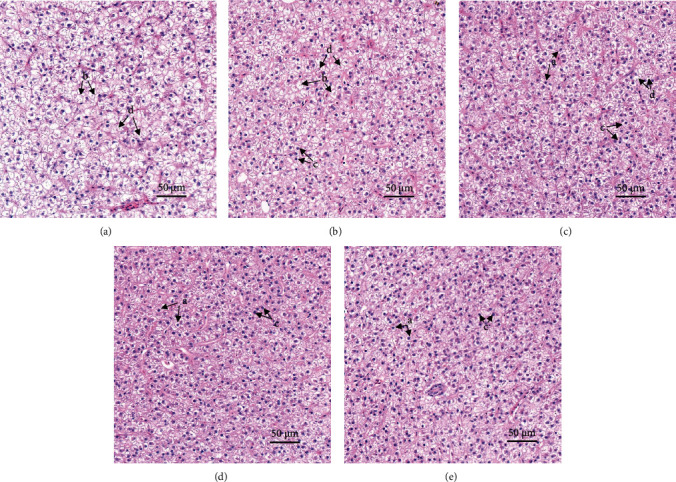
Photomicrographs of representative hematoxylin and eosin stained histological liver sections in 40x magnification: (A) CD (control, 43% carbohydrate) group; (B) 0.2B (0.2% betaine supplementation); (C) 0.4B (0.4% betaine supplementation); (D) 0.8B (0.8% betaine supplementation); and (E) 1.6B (1.6% betaine supplementation). Arrows indicate examples of (a) normal nucleus, regular and round nucleus located in the center of hepatocytes; (b) swollen hepatocytes, large amount of lipid accumulated in hepatocytes; (c) regular shaped hepatocytes; (d) hepatocytes without nucleus.

The histological structure of liver tissue in each group after H&E staining was evaluated. As shown in Figure 1, the volume of liver cells in the CD group was large, along with more anucleated hepatocytes. Dietary betaine supplementation significantly reduced the volume of hepatocytes, the number of non-nucleated cells, and the fatty accumulation. H&E staining results identified the regular structures of the liver in 0.8B and 1.6B groups.

Plasma ALT and AST activities and liver triglyceride content are shown in Table 4. The activity of plasma ALT and AST decreased with the increased level of betaine supplementation. Specifically, the enzyme activities in CD group and 0.2B group were significantly higher than those in the 0.4B group, and the enzyme activities in the 0.4B group were significantly higher than those in the 0.8B group and 1.6B group (*p* < 0.05). The liver triglyceride content also decreased with the increased level of betaine supplementation, and the CD group exhibited significantly higher level than the 0.4 B, 0.8B, and 1.6B groups (*p* < 0.05).

**Figure 2:**
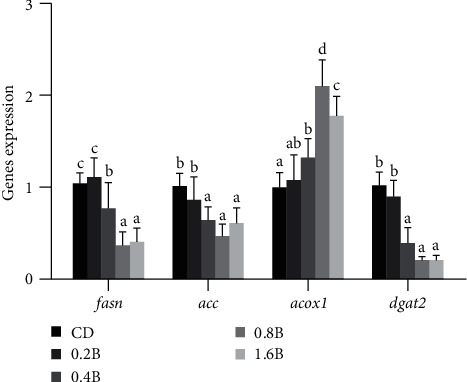
The gene expression of liver fat metabolism-related enzymes. CD: control; 0.2B: 0.2% betaine supplementation; 0.4B: 0.4% betaine supplementation; 0.8B: 0.8% betaine supplementation; and 1.6B: 1.6% betaine supplementation. Significant differences between groups are indicated by different letters (*p* < 0.05). ACC, acetyl COA carboxylase; ACOX, acyl COA oxidase 1; DGAT2, diacylglycerol acyltransferase 2; FAS, fatty acid synthase.

The expression of genes involved in liver fat metabolism is shown in Figure 2. The expression levels of *fasn*, *acc*, and *dgat2* related to lipogenesis decreased with the increased level of betaine supplementation. The expression level of *fas* in CD group and 0.2B group was significantly higher than that of *fas* in 0.4B group, and the expression level of *fas* in 0.4B group was significantly higher than that in 0.8B group and 1.6B group (*p* < 0.05). Similarly, the expression level of *acc* and *dgat2* in CD group and 0.2B group were significantly higher than those in 0.4 B, 0.8B, and 1.6B groups (*p* < 0.05). The expression level of *acox* related to lipolysis increased with the increased betaine addition level, with the highest expression level detected in 0.8B group, which was significantly higher than the other four groups (*p* < 0.05).

### 3.3. Taurine and Its Related Metabolites in Plasma

**Figure 3:**
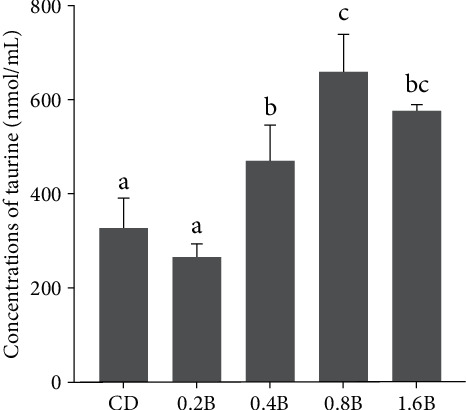
Concentrations of taurine in plasma of five groups. CD (control, 43% carbohydrate), 0.2B (0.2% betaine supplementation), 0.4B (0.4% betaine supplementation), 0.8B (0.8% betaine supplementation), and 1.6B (1.6% betaine supplementation). Values are presented as mean values with plus error bars (standard deviation). Significant differences between groups are indicated by different letters (*p* < 0.05).

**Figure 4:**
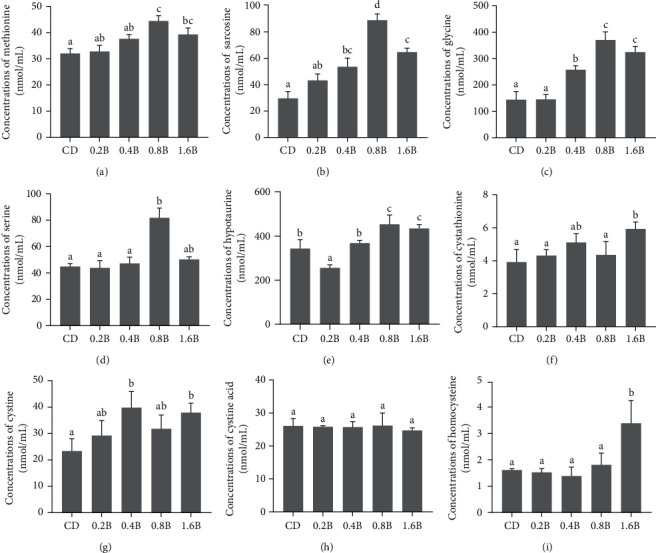
Concentrations of metabolites related to taurine synthesis in the plasma of five groups: (A) methionine; (B) sarcosine; (C) glycine; (D) serine; (E) hypotaurine; (F) cystathionine; (G) cystine; (H) cysteic acid; and (I) homocysteine. Values are presented as mean values of CD (control, 43% carbohydrate), 0.2B (0.2% betaine supplementation), 0.4B (0.4% betaine supplementation), 0.8B (0.8% betaine supplementation), and 1.6B (1.6% betaine supplementation) groups with plus error bars (standard deviation). Significant (*p*  < 0.05) differences between groups are indicated by different letters.

**Figure 5:**
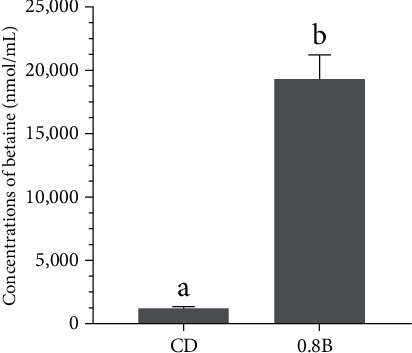
Betaine concentrations in plasma of CD (control, 43% carbohydrate) group and 0.8B (0.8% betaine supplementation) group.

Compared to the CD and 0.2B groups, the levels of taurine were significantly increased in the 0.4B, 0.8B, and 1.6B groups (*p* < 0.05). Among them, the level of taurine was the highest in 0.8B group and significantly higher than that in 0.4B group (*p* < 0.05, Figure 3). With the increase in dietary betaine supplementation from 0.2% to 0.8%, the levels of methionine, sarcosine, and glycine also increased, with the highest level detected in the 0.8B group, which were significantly higher than those in the 0.4B group (*p* < 0.05). However, the levels of these metabolites did not continue to increase but even decreased in the 1.6B group (Figure 4A–C). The level of serine in the 0.8B group was significantly higher than that in the CD, 0.2B and 0.4B groups (*p* < 0.05, Figure 4D). The levels of hypotaurine were significantly higher in the 0.8B and 1.6B groups than those in the other three groups (*p* < 0.05), with the highest level detected in the 0.8B group (Figure 4E). The levels of cystine and cystathionine were relatively high when the betaine addition levels were 0.4% and 1.6%. Compared to the other three groups, the level of cystathionine was significantly increased in the 1.6B group, while the levels of cystine in the 0.4B and 1.6B groups were only significantly higher than that in the CD group (*p* < 0.05, Figure 4F,G). The levels of cysteic acid were not significantly different among the five groups, but the levels of homocysteine were significantly higher in the 1.6B group than that in the other four groups (*p* < 0.05, Figure 4H,I). In addition, the level of betaine was significantly higher in the 0.8B group than that in the CD group (*p* < 0.05, Figure 5).

### 3.4. Gene Expressions of Key Enzymes Involved in Liver Taurine Metabolism

**Figure 6:**
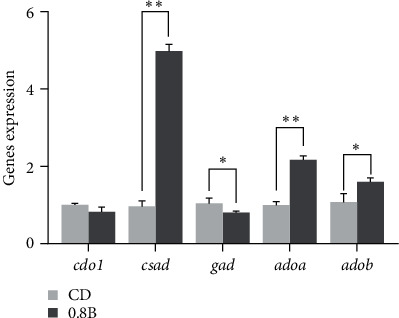
The genes expressions associated with taurine synthesis in liver of CD (control, 43% carbohydrate) and 0.8B (0.8% betaine supplementation) group.  ^*∗*^indicates a significant difference in data within the group (*p* < 0.05),  ^*∗∗*^indicates a very significant difference in data within the group (*p* < 0.01).

Considering the good results on the growth performance, liver histology, plasma taurine, and associated metabolites were all measured in 0.8% betaine supplementation group, the mRNA expressions levels of key enzymes in taurine metabolism were analyzed between the CD group and 0.8B group (Figure 6). Compared to CD group, the expression of *ado*-*a*, *ado-b*, and *csad* in 0.8B group were significantly upregulated (*p* < 0.05), while the expression of *gad* was significantly downregulated (*p* < 0.05). There was no significant difference in the expressions of *cod1* between the two groups (*p* > 0.05).

**Figure 7:**
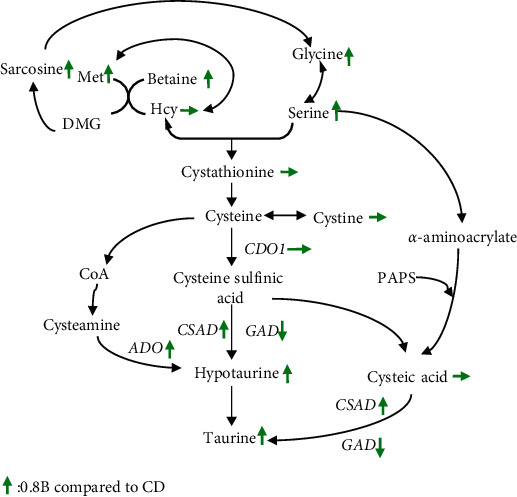
Overview of the metabolic pathway by which dietary betaine increase the synthesis of taurine in *M. amblycephala*. CD (control, 43% carbohydrate); 0.8B (0.8% betaine supplementation); ADO, cysteamine dioxygenase; ADOa, cysteamine dioxygenase a; ADOb, cysteamine dioxygenase b; CDO1, cysteine dioxygenase 1; CDO1, cysteine dioxygenase 1; CSAD, cysteine sulfinate decarboxylase; CoA, Coenzyme A; CSAD, cysteine sulfinate decarboxylase; DMG, dimethylglycine; GAD, glutamate decarboxylase; Hcy, homocysteine; Met: methionine; and PAPS, 3′-phosphoadenosine-5′-phosphosulfate. Bold arrows indicate the direction of the change in the concentration of a metabolite. Up arrow indicates a significant increase, down arrow indicates a significant decrease, and horizontal arrow indicates a nonsignificant change (0.8B compared to CD).

Thus, a path diagram of dietary betaine affecting taurine metabolism was drawn for *M. amblycephala* under high-carbohydrate conditions (Figure 7), which integrates the nutrient conversion pattern, betaine level, and taurine metabolism including the level of taurine and related metabolites, and the mRNA expressions of key enzymes.

### 3.5. Microbial Communities Analyzed by T-RFLP

**Figure 8:**
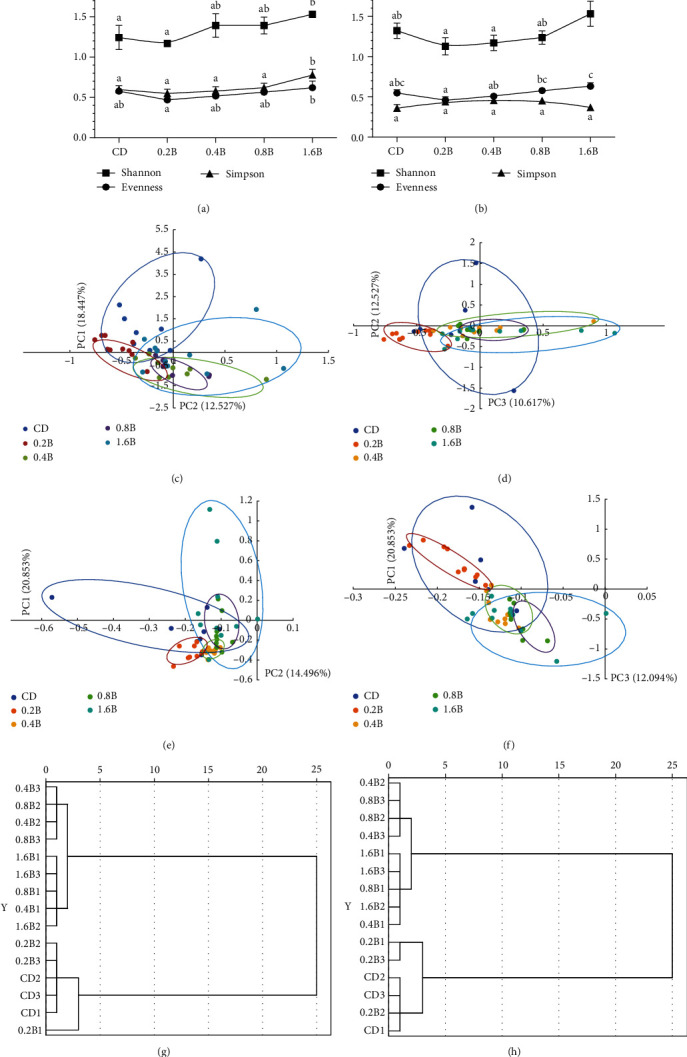
Microbial community indices (A and B), principal component analysis (C–F), and cluster analysis (G and H) of gut microbiota communities in five groups inferred by the *HaeIII* and *MspI* endonuclease digestion. CD (control, 43% carbohydrate); 0.2B (0.2% betaine supplementation); 0.4B (0.4% betaine supplementation); 0.8B (0.8% betaine supplementation); and 1.6B (1.6% betaine supplementation). Based on the principal component analysis results of T-RF composition, (A and B) panels show the microbial community indices of *HaeIII* and *MspI* digestion, respectively. Shannon (Shannon–Wiener index) and Simpson are measures of the diversity of a biological community; Evenness (Shannon evenness) means equitability of species relative abundances. (C and D) panels show the largest three weight scores of PC in *HaeIII* digestion results: PC1 explains 18.447% of the total variability of data, PC2 12.527%, and PC3 10.617%; (E and F) panels show the largest three weight scores of PC in *MspI* digestion results: PC1 explains 20.853%, PC2 14.496%, and PC3 12.094%. Based on the cluster analysis results of T-RF composition, (G and H) panels show the dendrograms of *HaeIII* and *MspI* digestion, respectively.

The effects of different betaine levels on the diversity and stability of gut microflora under high-carbohydrates condition were studied by the double enzyme digestion test of T-RFLP method. The digestion results of HaeIII showed that Shannon–Wiener index (H′), Simpson index (D′) and Evenness index (E′) all increased with the increase in betaine supplementation level (> 0.2%). Compared to CD and 0.2B groups, H′ was significantly higher in 1.6B group (*p* < 0.05), and *D′* and *E′* in the 1.6B group were also higher than those of 0.2B group (*p* < 0.05) (Figure 8A). The digestion results of *MspI* showed that the values of *H′* and *E′* both increased with the increase betaine supplementation level (> 0.2%). The H′ and E′ in 1.6B group were significantly higher than those in 0.2B and 0.4B groups (*p* < 0.05) (Figure 2B). The higher indexes including H′ and E′ of gut microflora in betaine supplementation groups (> 0.2%) indicated the more abundant, more stable, and more uniform microbial species in M. amblycephala gut. However, D′ values obtained by the two digestions were inconsistent, so the result for D′ was no longer valid in this experiment.

PCA was applied to identify patterns in the dataset, highlight similarities and differences in samples, and combine highly correlated variables into principal components (PCs) that replaced the original grouping variables. In the present study, three PCs with the largest weight scores (PC1, PC2, and PC3) at the T-RF level were chosen to reflect as much information as possible about the original variables. PCA scores in *HaeIII* and *MspI* datasets showed that the samples had high variability in CD and 0.2B groups, while the sample properties in 0.4B, 0.8B, and 1.6B groups were similar (Figure 8C–F). In the cluster analysis of T-RF, whether it was digested by *HaeIII* or *MspI*, the samples in CD and 0.2B groups could be divided into one category, while those in the 0.4B, 0.8B, and 1.6B groups were divided into another category (Figure 8G–H), which was consistent with the results of the PCA.

Combining the microbial community diversity index, PCA and cluster analysis, higher levels of betaine supplementation (> 0.4%) produced similar important transformations in the microbial communities and exerted a positive effect on gut microbes of *M. amblycephala* which were fed with high-carbohydrate diets.

## 4. Discussion

Dietary inclusion of carbohydrates at appropriate level showed the effects of saving protein, reducing feed cost, and aquaculture water pollution. However, excessive dietary carbohydrates caused metabolic disorders and stress responses in fish, and ultimately resulted in health damage [10, 11], as fish are considered to have a congenital “diabetic constitution” which is poorly tolerant to carbohydrates [40]. Betaine supplementation has been suggested to be an effective way to reverse damage caused by the high-carbohydrate diets in a variety of fish species including *M. megalobrama* [13, 41, 42]. Until now, the optimal level of betaine supplementation is still uncertain for *M. amblycephala* fed a high-carbohydrate diets, and the mechanism remains unclear. Therefore, we designed an experiment to identify the optimal amount of betaine supplementation with gradient level of betaine supplementation and proposed the hypothesis that betaine improved the liver health of *M. amblycephala* by increasing taurine synthesis.

### 4.1. Optimal Level of Betaine Supplementation in High-Carbohydrate Diets

Previous studies have found that dietary betaine supplementation could improve the growth performance of *M. amblycephala* [1], but the optimum supplementation amount is unknown. In *Oreochromis niloticus*, those fed with 0.5% betaine showed the best growth performance when fish were fed with diets supplemented with 0%, 0.5%, 1%, 1.5%, 2%, and 2.5% betaine [43]. Compared to diets with 0% and 1.5% betaine supplementation, growth performance was improved in deccan mahseer (*Tor khudree*) juveniles fed with 0.5% betaine supplementation [44]. Growth performance of *Marobrachium rosenbergii* was the best when the betaine content in the diet was 0.5% compared with 1% and 1.5% betaine supplementation level [45]. Similar results were found in rainbow trout [46] and broilers [47]. Thus, dietary betaine was less effective when supplementation was too high or too low, and only appropriate supplementation level was beneficial to fish growth. In the current study, 0.8B group had the highest WGR and SGR, and the lowest FCR, which suggested that 0.8% betaine supplementation level is beneficial to the growth and feed efficiency of *M. amblycephala*.

In recent years, plenty of literature have reported that betaine could exert good preventive and therapeutic effects on many liver diseases, such as chemical or drug-induced liver injury, nonalcoholic fatty liver disease, and alcoholic fatty liver disease [7]. Fish consuming high-carbohydrate diets typically exhibited fatty degeneration and accumulation of lipid droplets in the liver, accompanied by the reduction and disappearance of hepatocyte nuclei, at which point the liver was in a damaged state [4]. An indicator of liver health is maintaining liver cell normal morphology, including regular cells, large and round nuclei centrally located in the cell, and a small amount of lipid droplets in the cell. In previous studies, betaine supplementation at both 1.0% and 1.2% also demonstrated improvements in liver function [1, 20]. H&E staining results in this study showed that the addition of betaine could change the state of liver cells, including the decrease of liver cell volume and decreasing lipid droplet aggregation. Moreover, fewer abnormal cells were observed in the visual field of the 0.8B and 1.6B groups, which indicates better liver health.

Liver triglyceride (TG) content can reflect the degree of fat accumulation in the liver. Our results showed that betaine (> 0.4%) supplementation significantly reduced liver triglyceride content of *M. amblycephala*. Study has shown that adding mannan oligosaccharides (MOS) to a high-carbon diet improves lipid accumulation by inhibiting genes related to TG synthesis [48]. In this study, betaine promoted the expression of genes related to fatty acid synthesis, including *fas* and *acc*, and also increased the expression of *dgat2*, which promotes the conversion of fatty acids into triglycerides. At the same time, it was also found that the expression of *acox1*, which controls lipolysis, was inhibited. Therefore, betaine reduces hepatic lipid deposition of *M. amblycephala* by inhibiting fat synthesis and promoting lipolysis.

Alanine aminotransferase (ALT) and aspartate aminotransferase (AST) are present in hepatocytes. When the liver is damaged, the cell permeability increases, and these two enzymes are released, causing the enzyme activity or concentration to suddenly increase in the blood [49]. Mice fed with high-fat diet showed liver damage, and high serum ALT and AST levels, while intragastric infusion of betaine solution could significantly reduce the levels of these two enzymes [50]. Similar results were obtained in this experiment, dietary betaine supplementation (> 0.4%) significantly reduced the activity of ALT and AST in plasma of *M. amblycephala*, thereby alleviating liver cell damage.

Thus, supplementation of 0.8% and 1.6% betaine in the diet significantly improved the growth performance and liver health of *M. amblycephala*. However, from the perspective of ensuring feed quality and saving feed cost, the optimal supplemental amount of betaine is recommended not to exceed 0.8%.

### 4.2. Betaine Promoted Taurine Synthesis

Betaine administration (1%) was reported to affect the metabolism of sulfur-containing substances in rats and changed the level of taurine in the kidney, liver, and plasma [51, 52]. Little information was known about the changes in taurine caused by dietary betaine supplementations in fish species. In the current experiment, the contents of plasma taurine were also affected by dietary betaine supplementations in *M. amblycephala* fed with high-carbohydrate diets, and 0.8% betaine supplementation level in feed significantly increased plasma taurine content. In fact, three biological pathways of taurine synthesis have been identified in vertebrates [53]: cysteamine (CS) pathway, cysteine sulfinic acid (CSA) pathway, and cysteic acid (CA) pathway. Homocysteine and serine are the starting materials involved in the three pathways. As a methyl donor, betaine participated in the methionine cycle. Homocysteine received a methyl from betaine to produce methionine under the catalysis of betaine homocysteine methyltransferase (BHMT), and betaine lost a methyl to yield dimethylglycine (DMG) [54]. DMG was demethylated to sarcosine by dimethylglycine dehydrogenase, sarcosine was further metabolized to glycine by sarcosine dehydrogenase, and glycine was finally converted to serine by the serine hydroxymethyl transferase [54]. Supplemented betaine in diets could effectively increase the plasma betaine level in *M. amblycephala* [20]. Similarly, in this study, compared with the CD group, dietary betaine supplementation significantly increased the plasma betaine content in the 0.8B group. Therefore, the significant increase of methionine, sarcosine, glycine, and serine was reasonable as the metabolites of betaine. Serine could participate in taurine synthesis through the following two ways: (a) condensed with homocysteine to form cystathionine in a reaction mediated by cystathionine beta-synthase (C*β*S) [51], and cystathionine was converted to cysteine by cystathionine *γ*-lyase (C*γ*L) [54], then cysteine was further metabolized to hypotaurine and taurine consecutively [51, 52]. (b) Serine could be catalyzed to *α*-aminoacrylate by serine hydrolase, then *α*-aminoacrylate combined with 3′-phosphoadenosine-5′-phosphosulfate (PAPS) to produce cysteic acid, yielding taurine through the cysteic acid (CA) pathway [55]. In this study, although plasma serine levels were significantly increased when dietary betaine was supplemented, the levels of homocysteine, cysteine, cystathionine, and cysteic acid were not changed significantly. As reported in our early study, dietary betaine could decrease the mRNA expression of cystathionine *β* synthase in *M. amblycephala* fed with a high-carbohydrate diet [20], so the efficiency of serine and homocysteine conversion to cystathionine became lower. On the other hand, as the substrate of this reaction, the plasma homocysteine was not significantly changed. The sulfur in hypotaurine and taurine originates from cysteine and cysteic acid, while the sulfur in cysteine is derived from the cleavage of cystathionine and the conversion of cystine. The contents of cystathionine, cystine, and cysteic acid remained unchanged in the 0.8B group, while the contents of plasma hypotaurine and taurine were increased significantly in the present study, indicating that betaine might increase the contents of hypotaurine and taurine by enhancing the enzymatic action of key enzymes in CS, CA, and/or CSA pathway, rather than changing the key metabolite contents of cystathionine, cystine, and cysteic acid during the synthesis of taurine.

ADO, a key enzyme in the CS pathway, facilitates the synthesis of hypotaurine by cysteamine and oxygen [56–58]. Compared to CSAD, ADO was more widely distributed in fish, which means that the conversion of cysteamine to taurine might be a common pathway for taurine biosynthesis in fish [59]. By combining the transcriptome and genome databases of *M. amblycephala* with the sequences of ADO, two ADO gene sequences (ADO-a and ADO-b) were identified in the genome of *M. amblycephala*. In the present study, compared with the CD group, mRNA expression of both *ado-a* and *ado-b* were up-regulated in the 0.8B group. This result suggested that betaine might promote taurine synthesis by upregulation *ado* expression, although the contribution of ADO-a or ADO-b remains uncertain.

CDO and CSAD, two key enzymes in the CSA pathway, determine taurine biosynthesis ability [60, 61]. The enzyme activity or gene expression of *cdo1* and *csad* was often used to measure the ability of animals to synthesize taurine [61]. CDO1 is the vital enzyme in regulating cysteine concentration, and CSAD activity is the rate-limiting step in taurine biosynthesis [62]. CDO1-null and CSAD-null mouse were not able to synthesize hypotaurine/taurine by the CSA pathway and had quite low tissue taurine levels when they were fed a taurine-free diet [63]. In this study, compared with CD group, expression of *csad* in 0.8B group was upregulated while *cdo1* expression did not change significantly. Therefore, it was reasonable to speculate that betaine promoted taurine synthesis by upregulation *csad* expression. CSAD also catalyzed the conversion of cysteic acid in the CA pathway to taurine [64]. However, the ability of different fish to synthesize taurine varies greatly [65], whether CA pathway exists in *M. amblycephala* remained to be investigated. The CA pathway has been reported in bluegill (*Lepomis macrochirus*), red sea bream (*Pagrus major*), smelt fish (*Plecoglossus altivelis*), and rainbow trout (*Onchohynchus mykiss*) [66]. If the CA pathway was present in *M. amblycephala*, betaine might also promote taurine synthesis by promoting the CA pathway.

Thus, betaine can restore the reduction in tissue taurine content reduction caused by a high-carbohydrate diet by promoting endogenous taurine synthesis in *M. amblycephala*. The specific process is to upregulate ADO of the CS pathway and CSAD of the CSA pathway, and the CA pathway may also serve a function. Rather than changing the content of key metabolites (such as, cystathionine, cystine, or cysteine), betaine increases the synthesis of taurine by enhancing the activity of some key enzymes.

### 4.3. Betaine Stablized Intestinal Microbial Communities

The gut microbiota, as a main member of gut microecology, was an essential mediator in health and disease [67]. Gut microbiota is known to be closely related to the host's health, nutrition absorption, pathogen resistance, and enhancement of immune responses [68]. On the other hand, gut microbiota could be affected by the genotype, habitat, health condition, and diets of the host [69]. Betaine supplementation can stimulate the number and diversity of microbes in the intestine of tilapia and mouse [68, 70]. Similarly, in the present study, when betaine supplementation level was over 0.2%, the gut microbiota richness and stability index (*H′*), species evenness index (*E′*) both increased with the increased betaine supplementation level, indicated that within a certain range, dietary betaine could enhance the diversity, stability, and evenness of microbial communities in the gut of *M. amblycephala*. The possible reason is that betaine can be used as a source of methyl, carbon, and nitrogen for intestinal bacteria [68]. In addition, based on PCA of T-RF, the samples of the low-level betaine addition groups (CD and 0.2B) clustered into one category, and the samples of the high-level betaine addition groups (0.4B, 0.8B, and 1.6B) were clustered into another category. These results indicated that high dietary betaine supplementation (> 0.4%) induced similar and important shifts in the microbial communities of *M. amblycephala*. In conclusion, high betaine supplementation was more beneficial to the development of gut microbiota. According to the results of microbial diversity index, PCA and cluster analysis, the optimal dietary betaine supplementation level is 0.8%−1.6%.

## 5. Conclusion

According to growth performance and liver health, the optimum betaine supplementation level was 0.8% in high-carbohydrate (43%) diets for juvenile *M. amblycephala*. Dietary betaine supplementation exerted its protective roles in improving liver health of *M. amblycephala* via promoting de novo taurine synthesis (CS and CSA pathways) and stabilizing intestinal microbial communities. Further studies are needed to illustrate the specific mechanism by which dietary betaine induced de novo taurine synthesis and also define the main contributing bacteria species within the microbiota.

## Figures and Tables

**Table 1:** Composition and concentration of nutrients in five diets.

Ingredients (%)	Diets				
	CD	0.2B	0.4B	0.8B	1.6B
Soybean meal	38.50	38.50	38.50	38.50	38.50
Rapeseed meal	10.00	10.00	10.00	10.00	10.00
cottonseed meal	10.00	10.00	10.00	10.00	10.00
Wheat bran	5.00	5.00	5.00	5.00	5.00
Fish meal	5.00	5.00	5.00	5.00	5.00
Flour	21.60	21.60	21.60	21.60	21.60
Soybean oil	2.60	2.60	2.60	2.60	2.60
Methionine	0.10	0.10	0.10	0.10	0.10
Lysine	0.40	0.40	0.40	0.40	0.40
Monocalcium phosphate	2.00	2.00	2.00	2.00	2.00
Mineral and vitamin^a^	1.00	1.00	1.00	1.00	1.00
Choline chloride	0.20	0.20	0.20	0.20	0.20
Carboxymethyl cellulose	1.50	1.50	1.50	1.50	1.50
Betaine	0.00	0.20	0.40	0.80	1.60
Bentonite	1.60	1.40	1.20	0.80	0.00

Approximate composition (percentage of dry matter)					

Moisture	9.89	10.91	11.73	10.84	10.61
Crude protein	30.84	31.2	31.03	30.55	30.71
Crude ash	8.57	8.27	7.97	7.72	7.01
Crude lipid	7.00	7.26	7.53	7.19	6.96
Carbohydrates	43.47	43.36	42.32	42.46	43.07

Abbreviations: 0.2B, 0.2% betaine supplementation; 0.4B, 0.4% betaine supplementation; 0.8B, 0.8% betaine supplementation; 1.6B, 1.6% betaine supplementation; CD, control, 43% carbohydrate.

^a^Industry standard mineral and vitamin premix for herbivorous fish produced by the Beijing Yujing Biotechnology Co., Ltd.

**Table 2:** Primers designed for qPCR.

Target gene	Primer sequences
ADOa	F: 5′-CGAGCAGGCTTATGTGACCT-3′
	R: 5′-TGGAGACTTTCTTCGGTGGC-3′

ADOb	F: 5′-AAACCTAGAGACGGTGCCAG-3′
	R: 5′-ACCGATCTCAGCATCGAAGG-3′

CDO1	F: 5′-ATGGTGCAGAAGTCACAGCG-3′
	R: 5′-ACTGTAGAGGTGCAAGCTGAC-3′

CSAD	F: 5′-GCCGTAAGGTGGATTGCTTG-3′
	R: 5′-TGGCGCCACCTTTGATAGTC-3′

GAD	F: 5′-GGTGCCTTTGATCCTTTGATCG-3′
	R: 5′-TCGACTCCATTCAGCTTCCAC-3′

FAS	F: 5′-GCGTCTACATTGGAGTGAGTG-3′
	R: 5′-CTCGCTGGCATCCTGTCAT-3′

ACC	F: 5′-CCGAGGACAATTCTGAGGATG-3′
	R: 5′-TCATAAGTGCTGTCTGAACTGA-3′

DGAT2	F: 5′-GGACAGAGGCTACAGGATTCT-3′
	R: 5′-ACAGTGGCAAGCGGAAGT-3′

ACOX1	F: 5′-TGCGAAGGGTGTGTATCAAGA-3′
	R: 5′-TGGAACGACTCGGCTAACG-3′

Abbreviations: ACC, acetyl COA carboxylase; ADOa, cysteamine dioxygenase a; ADOb, cysteamine dioxygenase b; ACOX1, acyl COA oxidase 1; CDO1, cysteine dioxygenase 1; CSAD, cysteine sulfinate decarboxylase; DGAT2, diacylglycerol acyltransferase 2; FAS, fatty acid synthase; GAD, glutamate decarboxylase.

**Table 3:** Growth performance of *M. amblycephala* in five groups.

Items	CD	0.2B	0.4B	0.8B	1.6B
BWI (g)	6.79 ± 0.29	6.91 ± 0.05	6.69 ± 0.10	6.66 ± 0.01	6.68 ± 0.03
BWF (g)	14.85 ± 0.15^a^	15.11 ± 0.88^a^	15.20 ± 0.30^a^	16.18 ± 0.61^b^	15.91 ± 0.48^b^
WGR (%)	119.03 ± 11.59^a^	118.51 ± 11.05^a^	127.11 ± 1.25^ab^	142.88 ± 9.27^b^	138.13 ± 7.66^b^
SGR (%)	0.99 ± 0.07^a^	0.99 ± 0.06^a^	1.04 ± 0.01^a^	1.12 ± 0.05^b^	1.10 ± 0.04^b^
FCR	2.04 ± 0.16^b^	1.93 ± 0.01^b^	1.82 ± 0.07^ab^	1.63 ± 0.10^a^	1.79 ± 0.08^ab^
HSI (%)	1.53 ± 0.05^b^	1.44 ± 0.06^ab^	1.41 ± 0.02^a^	1.38 ± 0.01^a^	1.50 ± 0.01^b^
CF (%)	1.97 ± 0.05	1.98 ± 0.05	1.96 ± 0.09	2.02 ± 0.02	2.03 ± 0.03

*Note*: Values are presented as means of the entire group with standard deviation. Significant differences between groups are indicated by different letters (*p*  < 0.05).

Abbreviations: 0.2B, 0.2% betaine supplementation; 0.4B, 0.4% betaine supplementation; 0.8B, 0.8% betaine supplementation; 1.6B, 1.6% betaine supplementation; BWI, initial body weight; BWF, final body weight; CD, control; CF, condition factor; FCR, feed conversion ratio; HSI, hepatosomatic index; SGR, specific growth rate; WGR, weight gain rate.

**Table 4:** The activity of plasma ALT, AST, and liver triglyceride content.

Items	CD	0.2B	0.4B	0.8B	1.6B
ALT (U/L)	52.90 ± 5.11^c^	51.78 ± 2.90^c^	31.62 ± 1.80^b^	23.57 ± 2.16^a^	24.39 ± 4.43^a^
AST (U/L)	77.91 ± 3.08^c^	80.57 ± 4.87^c^	57.03 ± 5.46^b^	35.77 ± 4.33^a^	34.54 ± 3.95^a^
TG (mmol/gprot)	0.68 ± 0.09^b^	0.58 ± 0.09^ab^	0.50 ± 0.07^a^	0.44 ± 0.06^a^	0.46 ± 0.04^a^

*Note*: Values are presented as means of the entire group with standard deviation. Significant differences between groups are indicated by different letters (*p*  < 0.05).

Abbreviations: 0.2B, 0.2% betaine supplementation; 0.4B, 0.4% betaine supplementation; 0.8B, 0.8% betaine supplementation; 1.6B, 1.6% betaine supplementation; ALT, alanine aminotransferase; AST, aspartate aminotransferase; CD, control; TG, triglyceride.

## Data Availability

The data that support the findings of this study are available upon reasonable request from the corresponding author.

## References

[1] Adjoumani J.-J. Y., Wang K., Zhou M., Liu W., Zhang D. (2017). Effect of Dietary Betaine on Growth Performance, Antioxidant Capacity and Lipid Metabolism in Blunt Snout Bream Fed a High-Fat Diet. *Fish Physiology and Biochemistry*.

[2] FOOD and Agriculture Organization of the United Nations (FAO) (2022). The State of World Fisheries and Aquaculture, Towards Blue Transformation. https://www.fao.org/3/cc0461en/online/cc0461en.html.

[3] Kamalam B. S., Medale F., Panserat S. (2017). Utilisation of Dietary Carbohydrates in Farmed Fishes: New Insights on Influencing Factors, Biological Limitations and Future Strategies. *Aquaculture*.

[4] Prisingkorn W., Prathomya P., Jakovlić I., Liu H., Zhao Y.-H., Wang W.-M. (2017). Transcriptomics, Metabolomics and Histology Indicate that High-Carbohydrate Diet Negatively Affects the Liver Health of Blunt Snout Bream (*Megalobrama amblycephala*). *BMC Genomics*.

[5] Hemre G. I., Mommsen T. P., Krogdahl Å. (2002). Carbohydrates in Fish Nutrition: Effects on Growth, Glucose Metabolism and Hepatic Enzymes. *Aquaculture Nutrition*.

[6] Lin S.-M., Shi C.-M., Mu M.-M., Chen Y.-J., Luo L. (2018). Effect of High Dietary Starch Levels on Growth, Hepatic Glucose Metabolism, Oxidative Status and Immune Response of Juvenile Largemouth Bass, *Micropterus salmoides*. *Fish & Shellfish Immunology*.

[7] Wang T., Zhang N., Yu X.-B. (2021). Alleviates Adverse Metabolic Syndrome and Regulates Intestinal Microbiota Composition in Nile Tilapia (*Oreochromis niloticus*) Fed With High-Carbohydrate Diet. *British Journal of Nutrition*.

[8] Callet T., Hu H., Larroquet L. (2020). Exploring the Impact of a Low-Protein High Carbohydrate Diet in Mature Broodstock of a Glucose-Intolerant Teleost, the Rainbow Trout. *Frontiers in Physiology*.

[9] Boonanuntanasarn S., Kumkhong S., Yoohat K. (2018). Molecular Responses of Nile Tilapia (*Oreochromis niloticus*) to Different Levels of Dietary Carbohydrates. *Aquaculture*.

[10] Boonanuntanasarn S., Jangprai A., Kumkhong S. (2018). Adaptation of Nile Tilapia (*Oreochromis niloticus*) to Different Levels of Dietary Carbohydrates: New Insights From a Long Term Nutritional Study. *Aquaculture*.

[11] Liu C.-Z., He A.-Y., Ning L.-J. (2018). Leptin Selectively Regulates Nutrients Metabolism in Nile Tilapia Fed on High-Carbohydrate or High Fat Diet. *Frontiers in Endocrinology*.

[12] He C., Jia X., Zhang L. (2021). Berberine Can Ameliorate Glucose Metabolism Disorder of *Megalobrama amblycephala* Exposed to a High Carbohydrate Diet. *Fish Physiology and Biochemistry*.

[13] Wang F., Xu J., Jakovlic I., Wang W. M., Zhao Y. H. (2019). Dietary Betaine Reduces Liver Lipid Accumulation via Improvement of Bile Acid and Trimethylamine-N-Oxide Metabolism in Blunt-Snout Bream. *Food &*.

[14] Craig S. A. S. (2004). Betaine in Human Nutrition. *The American Journal of Clinical Nutrition*.

[15] Li S., Xu S., Zhao Y., Wang H., Feng J. (2020). Dietary Betaine Addition Promotes Hepatic Cholesterol Synthesis, Bile Acid Conversion, and Export in Rats. *Nutrients*.

[16] Fatahi S., Hoseini S. A., Sudagar M. (2013). The Effects of Dietary Betaine on the Growth Performance and Carcass Synthesis of Caspian Roach (*Rutilus Rutiluscaspicus*) Fingerlings. *Scientific Journal of Animal Science*.

[17] Jasour M. S., Wagner L., Sundekilde U. K. (2018). Fishmeal With Different Levels of Biogenic Amines in Aquafeed: Comparison of Feed Protein Quality, Fish Growth Performance, and Metabolism. *Aquaculture*.

[18] Kim M.-G., Lee C., Shin J., Lee B.-J., Kim K.-W., Lee K.-J. (2019). Effects of Fish Meal Replacement in Extruded Pellet Diet on Growth, Feed Utilization and Digestibility in Olive Flounder *Paralichthys olivaceus*. *Korean Journal of Fisheries and Aquatic Sciences*.

[19] Muhamad I. L., Setiawati M., Wiyoto W., Ekasari J. (2021). Dietary Supplementation of Betaine to Improve the Growth and Feed Utilization in Hybrid Grouper (*Epinephelus lanceolatus*♂ × *Epinephelus fuscoguttatus*♀) Juvenile. *Jurnal Akuakultur Indonesia*.

[20] Xu J., Wang F., Jakovlić I. (2018). Metabolite and Gene Expression Profiles Suggest a Putative Mechanism Through Which High Dietary Carbohydrates Reduce the Content of Hepatic Betaine in *Megalobrama amblycephala*. *Metabolomics*.

[21] Zhou S. N., Jiang M. W., Jiang X. M., Du X. X., Peng R. B., Han Q. X. (2019). Effects of Dietary Betaine on Feeding, Growth Performance, Tissue Nutritional Components and Digestive Enzyme Activities of *Hemifusus tuba* Gmelin. *Chinese Journal of Animal Nutrition*.

[22] Mohseni M., Saltanat N. L., Rastravan M. E., Golalipour Y. (2021). Effects of Betaine Supplementation in Plant-Protein-Based Diets on Growth Performance, Haemato-Immunological Parameters, Antioxidant Status and Digestive Enzyme Activities of Juvenile Caspian Trout (*Salmo trutta*, Kessler, 1877). *Aquaculture Nutrition*.

[23] Tiril S. U., Alagil F., Yagci F. B., Aral O. (2008). Effects of Betaine Supplementation in Plant Protein Based Diets on Feed Intake and Growth Performance in Rainbow Trout (*Oncorhynchus mykiss*). *Israeli Journal of Aquaculture Bamidgeh*.

[24] Koistinen V. M., Kärkkäinen O., Borewicz K. (2019). Contribution of Gut Microbiota to Metabolism of Dietary Glycine Betaine in Mice and in Vitro Colonic Fermentation. *Microbiome*.

[25] Sun H., Jiang W.-D., Wu P. (2020). Betaine Supplementations Enhance the Intestinal Immunity of on-Growing Grass Carp (*Ctenopharyngodon idella*): Partly Related to TOR and NF-*κ*B Signaling Pathways. *Aquaculture*.

[26] Storch K. J., Wagner D. A., Burke J. F., Young V. R. (1988). Quantitative Study in Vivo of Methionine Cycle in Humans Using [methyl-2H3]- and [1-13C] Methionine. *American Journal of Physiology-Endocrinology and Metabolism*.

[27] Stipanuk M. H., Coloso R. M., Garcia R. A. G., Banks M. F. (1992). Cysteine Concentration Regulates Cysteine Metabolism to Glutathione, Sulfate and Taurine in Rat Hepatocytes. *The Journal of Nutrition*.

[28] Lin S., Hirai S., Yamaguchi Y. (2013). 2013.Taurine Improves Obesity-Induced Inflammatory Responses and Modulates the Unbalanced Phenotype of Adipose Tissue Macrophages. *Molecular Nutrition & Food Research*.

[29] Tsuboyama-Kasaoka N., Shozawa C., Sano K. (2006). Taurine (2-Aminoethanesulfonic Acid) Deficiency Creates a Vicious Circle Promoting Obesity. *Endocrinology*.

[30] Aragão C., Teodósio R., Colen R. (2023). Supplementation to Plant-Based Diets Improves Lipid Metabolism in Senegalese Sole. *Animals*.

[31] Bai F., Niu X., Wang X., Ye J. (2021). Growth Performance, Biochemical Composition and Expression of Lipid Metabolism Related Genes in Groupers (*Epinephelus coioides*) Are Altered by Dietary Taurine. *Aquaculture Nutrition*.

[32] Liu H., Li H.-W., Xu Y.-J., Shi X.-G., Zhu Z.-C. (2006). Effects of Taurine on Growth and Nutritional Value of Carps. *Food Science and Technology International*.

[33] Zhang J., Che C., Cai M., Hu Y. (2022). Taurine Improves Health of Juvenile Rice Field Eel (*Monopterus albus*) Fed With Oxidized Fish Oil: Involvement of Lipid Metabolism, Antioxidant Capacity, Inflammatory Response. *Aquaculture Reports*.

[34] Zhou C., Ge X., Liu B., Xie J., Chen R., Ren M. (2015). Effect of High Dietary Carbohydrate on the Growth Performance, Blood Chemistry, Hepatic Enzyme Activities and Growth Hormone Gene Expression of Wuchang Bream (*Megalobrama amblycephala*) at Two Temperatures. *Asian-Australasian Journal of Animal Sciences*.

[35] Yu S., Olsen C. E., Marcussen J. (1997). Methods for the Assay of 1,5-Anhydro-d-Fructose and *α*-1,4-Glucan Lyase. *Carbohydrate Research*.

[36] Kanehisa M., Goto S. (2000). KEGG: Kyoto Encyclopedia of Genes and Genomes. *Nucleic Acids Research*.

[37] Liu H., Chen C., Gao Z. (2017). The Draft Genome of Blunt Snout Bream (*Megalobrama amblycephala*) Reveals the Development of Intermuscular Bone and Adaptation to Herbivorous Diet. *GigaScience*.

[38] Yin J., Liao S.-X., He Y. (2015). Dysbiosis of Gut Microbiota With Reduced Trimethylamine-N-Oxide Level in Patients With Large-Artery Atherosclerotic Stroke or Transient Ischemic Attack. *Journal of the American Heart Association*.

[39] Strong W. L. (2016). Biased Richness and Evenness Relationships Within Shannon–Wiener Index Values. *Ecological Indicators*.

[40] Wilson R. P. (1994). Utilization of Dietary Carbohydrate by Fish. *Aquaculture*.

[41] Fan C., Hu H., Huang X. (2022). Betaine Supplementation Causes an Increase in Fatty Acid Oxidation and Carbohydrate Metabolism in Livers of Mice Fed a High-Fat Diet: A Proteomic Analysis. *Foods*.

[42] Huang W., Hua Y., Wang F. (2022). Betaine and/or TMAO Affect Hepatic Lipid Accumulation and Glycometabolism of *Megalobrama amblycephala* Exposed to a High Carbohydrate Diet. *Fish Physiology and Biochemistry*.

[43] Luo Z., Tan X. Y., Liu X. J., Wen H. (2011). Effect of Dietary Betaine Levels on Growth Performance and Hepatic Intermediary Metabolism of GIFT Strain of Nile Tilapia *Oreochromis niloticus* Reared in Freshwater. *Aquaculture Nutrition*.

[44] Basade Y., Kohli M. P. S. (2011). Effect of Dietary Betaine on Growth Performance and Nutrient Utilization in Deccan Mahseer Tor Khudree (Sykes). *Indian Journal of Animal Nutrition*.

[45] Felix N., Sudharsan M. G. M. (2004). Effect of Glycine Betaine, a Feed Attractant Affecting Growth and Feed Conversion of Juvenile Freshwater Prawn *Macrobrachium rosenbergii*. *Aquaculture Nutrition*.

[46] Pinedo-Gil J., Tomás-Vidal A., Jover-Cerdá M., Tomás-Almenar C., Sanz-Calvo M.Á., Martín-Diana A. B. (2017). Red Beet and Betaine as Ingredients in Diets of Rainbow Trout (*Oncorhynchus mykiss*): Effects on Growth Performance. *Nutrient Retention and Flesh Quality.Archives of Animal Nutrition*.

[47] Leng Z., Fu Q., Yang X., Ding L., Wen C., Zhou Y. (2016). Fatty Acid -Oxidation as a Possible Mechanism for Fat-Reducing Effect of Betaine in Broilers. *Animal Science Journal*.

[48] Wang T., Wu H.-X., Li W.-J. (2022). Effects of Dietary Mannan Oligosaccharides (MOS) Supplementation on Metabolism, Inflammatory Response and Gut Microbiota of Juvenile Nile Tilapia (*Oreochromis niloticus*) Fed with High-Carbohydrate Diet. *Fish & Shellfish Immunology*.

[49] McGill M. (2016). The Past and Present of Serum Aminotransferases and the Future of Liver Injury Biomarkers. *EXCLI Journal*.

[50] Zhang W., Wang L.-W., Wang L.-K. (2013). Protects Against High-Fat-Diet-Induced Liver Injury by Inhibition of High-Mobility Group Box 1 and Toll-Like Receptor 4 Expression in Rats. *Digestive Diseases and Sciences*.

[51] Kim Y. C., Kwon D. Y., Kim J. H. (2014). Alterations in the Metabolomics of Sulfur-Containing Substances in Rat Kidney by Betaine. *Amino Acids*.

[52] Kim S. K., Kim Y. C. (2005). Effects of Betaine Supplementation on Hepatic Metabolism of Sulfur-Containing Amino Acids in Mice. *Journal of Hepatology*.

[53] Salze G. P., Davis D. A. (2015). Taurine: A Critical Nutrient for Future Fish Feeds. *Aquaculture*.

[54] Kim S. K., Choi K. H., Kim Y. C. (2003). Effect of Acute Betaine Administration on Hepatic Metabolism of S-Amino Acids in Rats and Mice. *Biochemical Pharmacology*.

[55] Kim S. K., Matsunari H., Takeuchi T. (2008). Comparison of Taurine Biosynthesis Ability Between Juveniles of Japanese Flounder and Common Carp. *Amino Acids*.

[56] Bella D., Kwon Y.-H., Hirschberger L., Stipanuk M. (2002). Post-Transcriptional Regulation of Cysteine Dioxygenase in Rat Liver. *Advances in Experimental Medicine and Biology*.

[57] Park E., Park S., Wang C., xu J., Lafauci G., Schuller-Levis G. (2002). Of Murine Cysteine Sulfinic Acid Decarboxylase and Its mRNA Expression in Murine Tissues. *Biochimica et Biophysica Acta (BBA)—Gene Structure and Expression*.

[58] Stipanuk M. H., Simmons C. R., Andrew Karplus P., Dominy J. E. (2011). Thiol Dioxygenases: Unique Families of Cupin Proteins. *Amino Acids*.

[59] Goto T., Matsumoto T., Takagi S. (2001). Distribution of the Hepatic Cysteamine Dioxygenase Activities in Fish. *Fisheries Science*.

[60] Stipanuk M. H., Ueki I., Dominy J. E., Simmons C. R., Hirschberger L. L. (2009). Cysteine Dioxygenase: A Robust System for Regulation of Cellular Cysteine Levels. *Amino Acids*.

[61] Wang X., He G., Mai K., Xu W., Zhou H. (2015). Ontogenetic Taurine Biosynthesis Ability in Rainbow Trout (*Oncorhynchus mykiss*). *Comparative Biochemistry and Physiology Part B: Biochemistry and Molecular Biology*.

[62] Wang Q., He G., Wang X., Mai K., Xu W., Zhou H. (2014). Dietary Sulfur Amino Acid Modulations of Taurine Biosynthesis in Juvenile Turbot (*Psetta maxima*). *Aquaculture*.

[63] Jurkowska H., Niewiadomski J., Hirschberger L. L. (2016). Downregulation of Hepatic Betaine: Homocysteine Methyltransferase (BHMT) Expression in Taurine-Deficient Mice Is Reversed by Taurine Supplementation in Vivo. *Amino Acids*.

[64] Martin W. G., Truex C. R., Tarka S. M., Hill L. J., Gorby W. G. (1974). The Synthesis of Taurine From Sulfate. VIII. A Constitutive Enzyme in Mammals. *Proceedings of Society Experimental Biology and Medicine*.

[65] El-Sayed A.-F. M. (2014). Dietary Taurine Supplementation Beneficial for Farmed Fish and Shrimp? A Comprehensive Review. *Reviews in Aquaculture*.

[66] Goto T., Mochizuki A., Hasumi F. (2002). Distribution and Activities of Enzymes Involved in Taurine Biosynthesis in the Liver of Fish. *Aquaculture Science*.

[67] Feng Q., Chen W.-D., Wang Y.-D. (2018). Gut Microbiota: An Integral Moderator in Health and Disease. *Frontiers in Microbiology*.

[68] He S., Zhou Z., Liu Y. (2012). Do Dietary Betaine and the Antibiotic Florfenicol Influence the Intestinal Autochthonous Bacterial Community in Hybrid Tilapia (*Oreochromis niloticus* ♀ × *O. aureus* ♂)?. *World Journal of Microbiology and Biotechnology*.

[69] Ni J., Yan Q., Yu Y., Zhang T. (2014). Factors Influencing the Grass Carp Gut Microbiome and Its Effect on Metabolism. *FEMS Microbiology Ecology*.

[70] Chen Q., Wang Y., Jiao F. (2020). Inhibits Toll-Like receptor 4 Responses and Restores Intestinal Microbiota in Acute Liver Failure Mice. *Scientific Reports*.

